# Growing Rod versus Posterior Spinal Fusion Treatment of Juvenile Idiopathic Scoliosis: Unique Characteristics and Surgical Outcomes

**DOI:** 10.3390/healthcare12040489

**Published:** 2024-02-18

**Authors:** Elizabeth M. Wacker, Lindsay Schultz, Nichole Leitsinger, Viral V. Jain, Peter F. Sturm

**Affiliations:** 1 University of Cincinnati Medical Center, Cincinnati, OH 45219, USA; wackerem@mail.uc.edu; 2 Cincinnati Children’s Hospital Medical Center, Cincinnati, OH 45229, USA; lindsay.schultz@cchmc.org (L.S.); nichole.leitsinger@cchmc.org (N.L.); viral.jain@cchmc.org (V.V.J.)

**Keywords:** scoliosis, juvenile idiopathic, growing rods, spinal fusion, treatment, outcomes, surgery

## Abstract

Progressive spinal curvature in juvenile idiopathic scoliosis (JIS) is challenging to treat. When conservative management fails, treatments include growing rods (GRs) or posterior spinal fusion (PSF). The purpose of this study is to compare the patient characteristics and outcomes of GR and PSF treatment of JIS. We performed a retrospective review of demographic, radiographic, and surgical data for all JIS patients requiring surgical treatment between 2012 and 2020. Patients who underwent any GR treatment were compared to PSF patients. A total of 36 patients (13 GR, 23 PSF) were reviewed. PSF patients had a larger pre-operative spinal height (*p* = 0.002), but similar pre-operative major curve magnitudes (*p* = 0.558). PSF treatment resulted in similar change in the T1-S1 length (*p* = 0.002), but a greater correction of the curve magnitude (*p* < 0.055) compared to GR patients. Eight patients initially treated with GRs later underwent definitive PSF treatment. This subset of patients had a greater spinal height before PSF (*p* = 0.006), but similar immediate post-PSF T1-S1 lengths (*p* = 0.437) and smaller changes in spinal height from PSF (*p* = 0.020) than primary PSF patients. At final follow-up, patients who underwent primary PSF versus PSF after GR had similar spinal heights (*p* = 0.842). The surgical intervention chosen to manage progressive JIS often differs based on patient characteristics. While this choice may impact immediate outcomes, the outcomes at final follow up are similar.

## 1. Introduction

Idiopathic scoliosis refers to a coronal plane curvature of the spine present without a known underlying etiology. The classification of idiopathic scoliosis is traditionally related to age at diagnosis—infantile (birth to 3 years and 11 months old), juvenile (4 years to 9 years and 11 months old), and adolescent (10 years to 17 years and 11 months old) [1]. Overall, juvenile idiopathic scoliosis is a rare condition with reports of only 4.5–21% of all idiopathic scoliosis fitting the classification of juvenile idiopathic scoliosis [2,3].

The progression of juvenile idiopathic scoliosis is variable and therefore, the approach to treatment is multifactorial. Natural history studies indicate that a small portion of juvenile idiopathic scoliosis curves treated with observation alone do not require further treatment because the curve magnitude remains stable or resolves [2,4,5,6,7,8]. However, it is reported that the majority of juvenile idiopathic scoliosis curves progress in magnitude enough to necessitate additional intervention [8,9]. In progressive curves, treatment strategies aim to maximize the natural growth of the spine while minimizing curve progression as both limitation of spinal growth and curve progression can have detrimental effects [10,11,12,13,14,15,16].

When curves progress despite observation, the next most conservative treatment option for juvenile idiopathic scoliosis is bracing. Understanding the outcomes of brace treatment for juvenile idiopathic scoliosis is complicated as there are variations in bracing protocols, compliance, and definitions of successful treatment. When taken as a whole, studies have shown that brace treatment leads to the maintenance or improvement of 28–51% of juvenile idiopathic scoliosis curves and can prevent surgical management of juvenile idiopathic scoliosis [2,4,5,6,8,17,18,19,20,21,22].

A subset of juvenile idiopathic scoliosis curves progress despite conservative management. These patients, who comprise approximately 27–56% of all juvenile idiopathic scoliosis patients, ultimately require surgical intervention [4,5,6,8,17,18,23]. A variety of surgical techniques have been used in the treatment of juvenile idiopathic scoliosis. The timing of surgery and the preferred operative technique remain difficult to decide given the wide variability in age, skeletal maturity, and rate of curve progression in patients with juvenile idiopathic scoliosis. As surgical treatment of juvenile idiopathic scoliosis has a large impact on the patient and their family, careful consideration of the surgical treatment options is paramount.

Traditionally, posterior spinal fusion was the mainstay of surgical intervention for juvenile idiopathic scoliosis [24]. There are several important considerations prior to posterior spinal fusion, including the skeletal maturity of the patient, the loss in spinal height growth, restriction of the thoracic cavity, and crankshaft phenomenon [2,12,25]. Surgical management with growing rods, either traditional or magnetically controlled, was developed with the hope of preserving the spinal height growth while managing the magnitude of curve progression. The use of growing rods requires the following set of considerations: increased clinic visits, repeat surgeries, implant complications, wound issues, unintended fusion, diminishing returns during subsequent lengthenings, and the need for a separate definitive treatment [2,24,26,27,28]. Despite the challenges, posterior spinal fusion and growing rods remain the most commonly used surgical interventions for progressive juvenile idiopathic scoliosis, yet few studies have compared these surgical techniques. The purpose of this study is to compare the pre-operative, intra-operative, and post-operative characteristics and outcomes of growing rod (traditional or magnetically controlled) versus posterior spinal fusion treatment of juvenile idiopathic scoliosis.

## 2. Materials and Methods

After approval from our hospital’s institutional review board, we began a retrospective review of all patients who underwent surgical treatment of juvenile idiopathic scoliosis at our pediatric hospital, which is a tertiary referral center. Our retrospective cohort study compared the pre-operative, intraoperative, and post-operative characteristics and outcomes of posterior spinal fusion versus growing rod treatment of juvenile idiopathic scoliosis. Potential participants were identified by searching the institution records for current procedural terminology (CPT) codes associated with growing rod implantation or posterior spinal fusion treatment. Patients were considered eligible for participation in the study if they were diagnosed with idiopathic scoliosis between 4 years and 9 years and 11 months old, and underwent posterior spinal fusion or placement of growing rods (either magnetically controlled or traditional) during the study period between 1 January 2012 and 31 December 2019. Patients were excluded if they had congenital, infantile, or adolescent scoliosis; pathology was identified in the spinal cord at any time; etiology of their scoliosis was identified at any time in their scoliosis treatment; they underwent vertebral body tethering; or curve progression did not necessitate surgical intervention.

Throughout treatment, all decisions were made at the discretion of the treating orthopedic surgeon. Demographic, clinical, and intraoperative data were collected via retrospective review of electronic medical records. Patients were followed from their preoperative visit until their final follow up visit at our institution. Radiographic measurements were obtained pre-operatively, in the immediate post-operative period, and at the final follow-up visit. Curve magnitudes were measured in the traditional way as the Cobb angle (reported in degrees) in the coronal plane and the major curve magnitude was defined as the largest Cobb angle of the measured curves. Spinal height was defined as the length (reported in centimeters) measured between a line placed at the most superior aspect of the T1 vertebra superior endplate and another line placed at the most inferior aspect of the S1 vertebra inferior endplate. All measurements were made with the annotation tools on PACS imaging software by a single, consistent physician.

Patients who underwent posterior spinal fusion treatment were compared to those that underwent placement of either traditional growing rods or magnetically controlled growing rods. The primary outcome of the study was the spinal height (measured as T1-S1 length) at the final follow up visit. Secondary outcomes of the study included the major coronal plane curve magnitude measured pre-operatively, in the immediate post-operative period, and at the final follow up visit and the changes in magnitude (reported in degrees) between these time points. The spinal height (measured as the T1-S1 length) measured pre-operatively and in the immediate post-operative period and the change in magnitude (reported) between these time points served as an additional set of secondary outcomes. Finally, the operative time (the time from incision to final closure), anesthesia time (the time from induction of general anesthesia to extubation of the patient), estimated blood loss (reported in milliliters), and length of hospital stay (reported in days) were considered additional secondary outcome measures.

A subset of the patients who were initially treated with growing rods underwent later definitive posterior spinal fusion at our institution. Definitive posterior spinal fusion was pursued when patients had progression of their spinal curvature despite growing rod treatment, were no longer successfully lengthening through their growing rods, or had maximized the extent of their growing rod construct and were near skeletal maturity. The patients who initially underwent growing rod treatment and were transitioned to definitive posterior spinal fusion at a later time point were considered a subgroup at the time of their definitive posterior spinal fusion. This subgroup was compared to the group of patients who underwent primary posterior spinal fusion as their initial and only treatment.

Descriptive statistics were used to summarize the demographic data, intraoperative characteristics, and pre-operative, immediate post-operative, and final visit radiographic measurements. Mann–Whitney U tests were used to compare the demographic data and pre-operative, intra-operative, and final post-operative data and measurements. The data were analyzed using IBM SPSS Statistics (Version 28, Armonk, NY, USA). The statistical significance was set at 0.05 for all analyses.

## 3. Results

A total of 36 patients at our institution met the criteria to undergo surgical treatment of progressive juvenile idiopathic scoliosis during the study period. Ultimately 36.1% (13/36) of these patients were surgically treated with growing rods (either traditional or magnetically controlled), while 63.9% (23/36) were surgically managed with posterior spinal fusion (see Table 1). The overall average age of all patients was 10.2 years old at the time of surgical intervention, while the average age of those treated with growing rods and posterior spinal fusion was 9.3 years old and 10.7 years old, respectively (see Table 1). Only 8.3% (3/36) of the patients were male and all of these patients were treated with growing rods.

Surgical management of juvenile idiopathic scoliosis with growing rod placement consisted of implantation of either traditional growing rods or magnetically controlled growing rods. The majority of growing rod treatments utilized magnetically controlled growing rods (76.9%, 10/13). Regardless of the type of growing rod that was used, surgery involved limited dissection, pedicle screw insertion, and fusion at the upper and lower few vertebrae of the construct with submuscular introduction of the growing rods prior to seating the rods in the pedicle screws. The upper instrumented vertebra in the growing rod constructs was typically T2 or T3. Only one construct utilized T4 as the upper instrumented vertebra. The cranial local fusion typically spanned three vertebrae (for example, from T2 to 4), though four of the constructs only involved two vertebrae (for example, from T3 to 4). The lowest instrumented vertebra ranged from L2 to L4. The caudal local fusion was typically only two vertebrae (only five of these local fusions involved three vertebrae).

**Figure 1:**
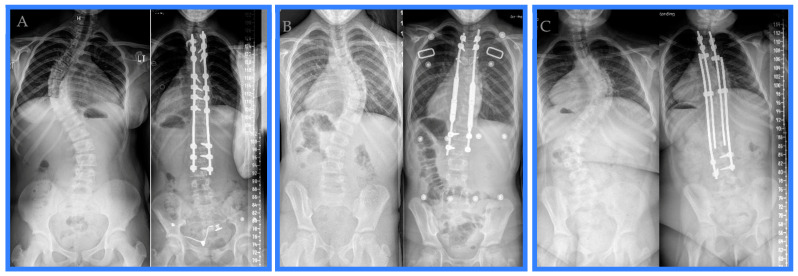
Pre-operative and first post-operative appointment radiographs exemplifying a (**A**) posterior spinal fusion, (**B**) magnetically controlled growing rod, and (**C**) traditional growing rod treatment of juvenile idiopathic scoliosis during the study period.

Variations existed among the posterior spinal fusions performed for surgical management of juvenile idiopathic scoliosis during the study period. The lengths of the posterior spinal fusion constructs ranged from 11 to 14 vertebrae. The construct length was 13 vertebrae 40% of the time, 14 vertebrae in 26% of posterior spinal fusions, 11 vertebrae 17% of the time, and 12 vertebrae in the remaining 17% of cases. The upper instrumented vertebra was instrumented with a hook over the transverse process in all but one posterior spinal fusion. In the case that did not utilize hooks on the upper instrumented vertebra, pedicle screws were used as the most superior instrumentation. T3 was the most common upper instrumented vertebra (70%). The next most common upper instrumented vertebra was T2 (26%), and only one construct (4%) utilized T4 as the upper instrumented vertebra. The lowest instrumented vertebra was more variable, with ten constructs utilizing L3 (43%), five stopping at L4 (22%), four ending at L1 (17%), two constructs going to T12 (9%), and two constructs finishing at L2 (9%). Two constructs utilized a combination of pedicle screws and sublaminar wires to anchor the rods; otherwise, the posterior spinal fusion constructs were dual rods affixed to the spine with a mix of intermittent uniaxial and polyaxial pedicle screws. Examples of the posterior spinal fusion, the magnetically controlled growing rod, and the traditional growing rod constructs are provided (see Figure 1).

At the time of surgery, patients undergoing placement of growing rods were significantly younger (*p* = 0.001), shorter (*p* = 0.001), and weighed less (*p* = 0.002) than those being treated with posterior spinal fusion (see Table 1). The average pre-operative major curve magnitude (Cobb angle) did not differ between patients treated with growing rods and posterior spinal fusion (*p* = 0.558). The pre-operative spinal height (T1-S1 length) was significantly shorter in the growing rod group (*p* = 0.002) (see Table 1).

Growing rod placement surgery took a significantly shorter amount of operative time compared to posterior spinal fusion surgery (*p* = 0.001) and resulted in less estimated blood loss during surgery (*p* < 0.001) (see Table 1). Similarly, the total time under general anesthesia was significantly shorter for the patients treated with growing rods than those treated with posterior spinal fusion (*p* < 0.001) (see Table 1). The time that anesthesia independently cared for the patient was similar between the groups (*p* = 0.515), which indicates that the operative time (time from incision to final closure) for a given surgical treatment had the greatest impact on the total time under general anesthesia for that procedure (see Table 1). The posterior spinal fusion cohort had a longer length of stay in the hospital after surgery compared to the growing rod treatment group (*p* = 0.02) (see Table 1).

As a result of surgical intervention, the major curve magnitude was corrected more significantly in the posterior spinal fusion treatment group than the growing rod treatment group. This is evidenced by both a smaller average Cobb angle in the immediate post-operative period (*p* < 0.001) and a larger change in the Cobb angle for the patients who were treated with posterior spinal fusion, which trends towards significance (*p* = 0.055) (see Table 2). The average spinal height in the immediate post-operative period was significantly different between the two cohorts (*p* = 0.002), with patients who underwent posterior spinal fusion demonstrating a greater average spinal height. However, the difference in the immediate post-operative T1-S1 length and the pre-operative T1-S1 length as a result of surgery was not significant between the growing rod and posterior spinal fusion treatment groups (*p* = 0.548) (see Table 2). This is likely a reflection of the shorter pre-operative spinal height in the growing rod cohort and indicates that growing rod treatment and posterior spinal fusion treatment have a similar effect in lengthening the spinal height during the index surgery.

Of the patients who were initially treated with growing rods, 61.5% (8/13) underwent subsequent definitive posterior spinal fusion at our institution by the time of final data collection. These patients who were initially treated with growing rods had their definitive posterior spinal fusion an average of 3.8 years after their initial growing rod placement surgery (average: 3.8 years ± 1.3 years). This subset of patients demonstrated a longer pre-operative T1-S1 length prior to their posterior spinal fusion when compared to the patients who underwent posterior spinal fusion as an initial, sole treatment (*p* = 0.006) (see Table 3). The spinal height in the immediate post-operative period following definitive posterior spinal fusion after initial growing rod treatment was similar to the immediate post-operative period T1-S1 length in the primary posterior spinal fusion cohort (*p* = 0.437). The change in spinal height as the result of the later definitive posterior spinal fusion after growing rod treatment was significantly less than the change in spinal height as the result of a primary posterior spinal fusion in the cohort of patients who underwent posterior spinal fusion as a singular surgical intervention (*p* = 0.02) (see Table 3). This indicates that definitive posterior spinal fusion after the initial growing rod treatment has a less significant effect in lengthening the spinal height compared to primary posterior spinal fusion as a sole surgical treatment. At final follow up, the spinal height was similar regardless of whether the patient underwent primary posterior spinal fusion alone or first underwent growing rod treatment followed by subsequent posterior spinal fusion (*p* = 0.842) (see Table 3). On average, the final follow-up visit was 3.4 years after the patient underwent initial surgical treatment.

## 4. Discussion

Progressive juvenile idiopathic scoliosis that is unresponsive to conservative management efforts necessitates surgical treatment, usually growing rod placement or posterior spinal fusion. Our study of 36 patients treated surgically for progressive juvenile idiopathic scoliosis evaluated the pre-operative, intra-operative, and post-operative characteristics and outcomes of treatment with growing rods versus posterior spinal fusion. We defined the characteristics of patients most likely to receive a given surgical intervention. Patients who are younger, are shorter, weigh less, and have a shorter pre-operative T1-S1 spinal height are more likely to be treated with growing rods (either magnetically controlled or traditional), while the magnitude of the pre-operative major curve does not impact the surgical technique choice by the treating orthopedic surgeon. The characteristics of the patients in our study are similar to other cohorts of juvenile idiopathic scoliosis patients differentially treated with growing rods or posterior spinal fusion [29,30,31].

We analyzed the intraoperative and immediate post-operative consequences of treating progressive juvenile idiopathic scoliosis with growing rods versus posterior spinal fusion. Treatment with posterior spinal fusion requires a longer operative time and time under general anesthesia and leads to higher blood loss and a longer length of hospital stay. The benefits of posterior spinal fusion in the immediate post-operative period are a greater improvement in the major curve magnitude and a longer spinal height. Studies by Pawelek et al. and Keil et al. comparing treatment with posterior spinal fusion and growing rod placement yielded similar findings that posterior spinal fusion has a greater impact on major curve correction in the immediate post-operative period than implantation of growing rods [30,32].

Understanding the pre-operative, intra-operative, and immediate post-operative characteristics and outcomes is important in the comprehensive treatment of the juvenile idiopathic scoliosis patient, though understanding the persistent effects of surgical intervention is more pertinent. Our study found that patients initially treated with growing rods had a pre-operative spinal height at the time of their definitive posterior spinal fusion that was, on average, 4.3 cm longer than the initial spinal height of patients who underwent index posterior spinal fusion. The cohorts studied by Pawelek et al. and Keil et al. similarly demonstrated an increased spinal height (T1-S1 length) during growing rod treatment, resulting in a longer T1-S1 height at the time of posterior spinal fusion [30,31,32]. Our findings, in conjunction with the findings of previous studies, suggest that the goal of growing rod treatment to allow for longitudinal growth of the spine prior to posterior spinal fusion is met.

Ultimately, the impact of growing rod treatment on spinal height is not maintained through definitive posterior spinal fusion. That is to say, the spinal height (T1-S1 length) following posterior spinal fusion does not differ between patients treated primarily with posterior spinal fusion alone compared to those initially treated with growing rods followed by subsequent posterior spinal fusion. This is true in both the immediate post-operative period following posterior spinal fusion and at final follow up. This corroborates the report of Pawelek et al. that the overall percentage gain in spinal height (T1-S1 length) after secondary posterior spinal fusion does not significantly differ from that of those treated only with primary posterior spinal fusion [30]. Though Mackey et al. compared three surgical treatment groups (vertebral body tethering, magnetically controlled growing rods, and posterior spinal fusion) for management of juvenile idiopathic scoliosis, they similarly found no significant difference in the final T1-S1 length between any of the treatment groups [29].

The proposed benefits of growing rod placement for progressive juvenile idiopathic scoliosis curves include maintenance of longitudinal spine growth and control of the progression of spinal curvature. This study implies that the proposed benefits of growing rod treatment do not persist beyond the definitive posterior spinal fusion. Though the average spinal height of patients treated with growing rods increases throughout this treatment and is overall longer at time of posterior spinal fusion than the pre-operative T1-S1 length of patients who undergo primary posterior spinal fusion, the change in spinal height as a result of the posterior spinal fusion surgery is smaller in the cohort initially treated with growing rods, and the average T1-S1 length at final follow up of these two cohorts is similar. Presumably, stiffness or auto-fusion develops during growing rod treatment, which inhibits the magnitude of the increased axial length as a result of posterior spinal fusion after growing rod treatment. This suggestion is supported by prior studies that have evaluated diminishing returns on repeated growing rod lengthenings [26].

The current research must be interpreted within the context of the study design. Limitations to our study include that we performed a retrospective cohort study with the possibility of selection bias. We aimed to minimize this by applying our inclusion and exclusion criteria to a consecutive series of juvenile idiopathic scoliosis patients undergoing surgical treatment. As juvenile idiopathic scoliosis is a relatively rare condition and surgical treatment and is only appropriate in a subset of patients, sample sizes are inevitably limited, as is the case in this study. The statistical tests utilized for this study were chosen to minimize the effect of the small size of the treatment groups. Evaluating the Cobb angle and T1-S1 length measurements across consecutive radiographs makes the comparison of the measurements subject to rotational differences between images, though the differences in positioning are expected to be minimal, as all images were obtained in a standard way at the same institution. We did not assess for the crankshaft phenomenon, a known consequence of posterior spinal fusion at a young age, as numerical assessment of this characteristic is technically difficult to obtain and compare. Finally, further prospective investigations would be necessary to evaluate any functional outcomes of growing rod treatment versus posterior spinal fusion treatment, as our study did not assess functional outcome measurements.

## 5. Conclusions

Surgical management decisions for juvenile idiopathic scoliosis require careful consideration by the treating orthopedic surgeon. As suggested in this study, posterior spinal fusion surgery is a more extensive operation, with many immediate operative and post-operative considerations; however, the benefits include a single surgical intervention with strong correction of the major curve magnitude and a similar ultimate spinal height. Growing rod treatment results in multiple subsequent interventions and the ultimate need for definitive surgery, which likely balances the benefits of less extensive initial surgical interventions and immediate post-operative needs. Growing rod treatment is still an important consideration in very young juvenile idiopathic scoliosis patients with a quickly progressive curve, as preservation of thoracic cavity development is more paramount. Balancing all contributing factors can be challenging. Our study identifies differences between growing rod treatment and posterior spinal fusion management for juvenile idiopathic scoliosis. As such, pediatric orthopedic surgeons can integrate this information into their treatment framework. We suggest a strong consideration of initial posterior spinal fusion treatment when surgical intervention for progressive juvenile idiopathic scoliosis becomes necessary.

## Figures and Tables

**Table 1:** Comparison of pre-operative demographic characteristics, pre-operative radiographic measurements, and operative characteristics.

	Growing Rods (*n* = 13, 36.1%)	Posterior Spinal Fusion (*n* = 23, 63.9%)	*p* Value
**Pre-Operative Demographics**			
Age (years)	9.3 ± 1.6	10.7 ± 0.6	0.001 *
Height (cm)	137.1 ± 11.8	150 ± 7.3	0.001 *
Weight (kg)	36.1 ± 11.7	50.5 ± 12.9	0.002 *
**Pre-Operative Measurements**			
Cobb Angle (degrees)	61.5 ± 13.4	59.9 ± 9.3	0.558
T1-S1 Length (cm)	33.3 ± 4.0	37.2 ± 2.7	0.002 *
**Operative Characteristics**			
Operative time (minutes)	176.5 ± 36.5	238.7 ± 36.9	0.001 *
Total Anesthesia Time (minutes)	269.9 ± 38.8	328 ± 39	<0.001 *
Independent Anesthesia Time (minutes)	93.4 ± 21.3	89.3 ± 11.7	0.515
Estimated Blood Loss (mL)	152.7 ± 146.8	556.3 ± 306.1	0.001 *
Length of Stay (days)	2.5 ± 1.2	3.5 ± 0.9	0.02 *

* Indicates a statistically significant finding.

**Table 2:** Comparison of pre-operative, immediate post-operative, and final follow up radiographic measurements in patients treated with growing rods versus posterior spinal fusion.

	Growing Rods (*n* = 13, 36.1%)	Posterior Spinal Fusion (*n* = 23, 63.9%)	*p* Value
**Pre-Operative**			
Cobb Angle (degrees)	61.5 ± 13.4	59.9 ± 9.3	0.558
T1-S1 Length (cm)	33.3 ± 4.0	37.2 ± 2.7	0.002 *
**Immediate Post-Operative**			
Cobb Angle (degrees)	22 ± 6.7	12.1 ± 7.0	<0.001 *
Change in Cobb Angle (degrees)	39.5 ± 12.4	47.8 ± 8	0.055
T1-S1 Length (cm)	37.8 ± 4.6	42.8 ± 3.6	0.002 *
Change in T1-S1 Length (cm)	4.5 ± 2.1	5.6 ± 3.3	0.548

* Indicates a statistically significant finding.

**Table 3:** Comparison of pre-operative, immediate post-operative, and final follow up radiographic measurements for patients treated with later, definitive posterior spinal fusion after initial growing rod treatment versus primary posterior spinal fusion.

	PSF after Initial Treatment with GR (*n* = 8, 22.2%)	Primary PSF (*n* = 23, 63.9%)	*p* Value
Pre-PSF T1-S1 Length (cm)	41.3 ± 3.8	37.2 ± 2.7 (as in Table 2)	0.006 *
Immediately Post-Op PSF T1-S1 Length (cm)	44.3 ± 2.6	42.8 ± 3.6 (as in Table 2)	0.437
Change in T1-S1 Length from PSF (cm)	3.0 ± 3.3	5.6 ± 3.3 (as in Table 2)	0.02 *
Final Follow-Up T1-S1 Length (cm)	43.5 ± 4.2	43.1 ± 3.5	0.842

* Indicates a statistically significant finding.

## Data Availability

The raw data supporting the conclusions of this article can be made available by the authors on request.
